# Genetic Susceptibility to Arrhythmia Phenotypes in a Middle Eastern Cohort of 14,259 Whole-Genome Sequenced Individuals

**DOI:** 10.3390/jcm13041102

**Published:** 2024-02-15

**Authors:** Fatima Qafoud, Mohamed Elshrif, Khalid Kunji, Asma Althani, Amar Salam, Jassim Al Suwaidi, Nidal Asaad, Dawood Darbar, Mohamad Saad

**Affiliations:** 1 College of Health Sciences, Qatar University, Doha P.O. Box 2713, Qatar; fkafood@hotmail.com (F.Q.); aaja@qu.edu.qa (A.A.); 2 Qatar Computing Research Institute, Hamad Bin Khalifa University, Doha P.O. Box 5825, Qatar; melshrif@hbku.edu.qa (M.E.); kkunji@hbku.edu.qa (K.K.); 3 Department of Cardiology, Al-Khor Hospital, Hamad Medical Corporation, Doha P.O. Box 3050, Qatar; asalam@hamad.qa; 4 Heart Hospital, Hamad Medical Corporation, Doha P.O. Box 3050, Qatar; jalsuwaidi@hamad.qa (J.A.S.); nasaad@hamad.qa (N.A.); 5 Division of Cardiology, Department of Medicine, University of Illinois Chicago, Chicago, IL 60612, USA; darbar@uic.edu

**Keywords:** arrythmia, atrial fibrillation, cardiomyopathy, Middle East, genomics, diversity, whole-genome sequencing, diverse populations

## Abstract

**Background:** The current study explores the genetic underpinnings of cardiac arrhythmia phenotypes within Middle Eastern populations, which are under-represented in genomic medicine research. **Methods:** Whole-genome sequencing data from 14,259 individuals from the Qatar Biobank were used and contained 47.8% of Arab ancestry, 18.4% of South Asian ancestry, and 4.6% of African ancestry. The frequency of rare functional variants within a set of 410 candidate genes for cardiac arrhythmias was assessed. Polygenic risk score (PRS) performance for atrial fibrillation (AF) prediction was evaluated. **Results:** This study identified 1196 rare functional variants, including 162 previously linked to arrhythmia phenotypes, with varying frequencies across Arab, South Asian, and African ancestries. Of these, 137 variants met the pathogenic or likely pathogenic (P/LP) criteria according to ACMG guidelines. Of these, 91 were in ACMG actionable genes and were present in 1030 individuals (~7%). Ten P/LP variants showed significant associations with atrial fibrillation *p* < 2.4 × 10^−10^. Five out of ten existing PRSs were significantly associated with AF (e.g., PGS000727, *p* = 0.03, OR = 1.43 [1.03, 1.97]). **Conclusions:** Our study is the largest to study the genetic predisposition to arrhythmia phenotypes in the Middle East using whole-genome sequence data. It underscores the importance of including diverse populations in genomic investigations to elucidate the genetic landscape of cardiac arrhythmias and mitigate health disparities in genomic medicine.

## 1. Introduction

Cardiac arrhythmia, characterized by irregular heartbeats, encompasses a spectrum of conditions ranging from benign to life-threatening. The etiology of cardiac arrhythmias is complex, encompassing genetic predispositions, environmental influences, the remodeling of cardiac tissue, and lifestyle factors [1]. Advancements in genomic research have significantly contributed to unraveling the genetic foundations of arrhythmias, shedding light on their complex pathophysiology and paving the way for novel therapeutic approaches [2]. Atrial fibrillation (AF), the most common sustained arrhythmia in clinical practice, is associated with increased risks of heart failure, dementia, stroke, and death [3,4]. Risk factors for AF include diabetes, obesity, and hypertension [5,6]. Genetic variability plays a crucial role in the etiology of AF, with studies showing heritability rates and the involvement of multiple genes [7]. Large-scale genome-wide association studies (GWASs) have identified over a hundred loci associated with AF [8].

Despite these advancements, the majority of GWASs for AF have been conducted in populations of European descent, with a lack of representation from Middle Eastern populations [9,10]. The genetic architecture of AF in Middle Eastern cohorts, particularly considering both rare and common variants, remains unclear. Polygenic risk scores (PRSs) have shown promise in discriminating individuals at an increased risk of developing arrhythmia [11,12]. However, the applicability of PRSs developed in predominantly white cohorts to other ancestries, including Middle Eastern populations, has been a subject of debate [13]. Our study explores the genetic landscape of cardiac arrhythmias and AF in the Middle Eastern population using data from Qatar Biobank (QBB), examining rare and common variants through whole-genome sequencing (WGS), and evaluating the performance of PRSs. This research aims to contribute valuable insights into the genetic architecture of arrythmias and AF in a region that has been notably understudied, potentially addressing health disparities, and advancing our understanding of arrhythmia-related phenotypes.

## 2. Material and Methods

### 2.1. Study Cohort

The QBB longitudinal population-based cohort study aims to recruit a population sample (*N* = 60,000) of permanent Qatari residents and follow up with them every 5 years. Individuals are eligible to participate in the study if they are Qatari nationals or long-term residents (≥15 years living in Qatar) aged 18 years and older. The study covers extensive baseline sociodemographic data, clinical and behavioral phenotypic data, biological samples (i.e., blood, urine, saliva, DNA, RNA, viable cells, and others), as well as clinical biomarkers and omics data (i.e., genomics, transcriptomics, proteomics, metabolomics, etc.) [14]. All QBB participants signed an informed consent form prior to their participation. QBB study protocol ethical approval was obtained from the Hamad Medical Corporation Ethics Committee (MRC/12113/2012) on 15 August 2012 and continued with the QBB Institutional Review Board (IRB) (Full Board-2017-QF-QBB-RES-ACC-0075-0023) from 18 June 2017 onwards, and it is renewed on an annual basis. For this study, all available WGS data from 14,669 Qatari nationals representing individuals with Middle Eastern ancestry were used.

### 2.2. Whole-Genome Sequencing and Quality Control

The DNA extraction was conducted from Buffy’s coat using the Hamilton Chemagic STAR DNA Buffy Coat2k kit procedure based on automated magnetic bead technology (PerkinElmer; Massachusetts, USAart. No. CMG1792). Whole-genome sequencing was completed by integrated genomic services at Sidra Medicine. The Illumina TruSeq DNA Nanokit was used to create whole-genome libraries from 150 ng of DNA (San Diego, CA, USA). Illumina’s HiSeq X Ten was used to carry out genome sequencing on the HiSeq X Ten (San Diego, CA, USA) using the manufacturer’s suggested procedure. The WGS average read depth was 30x. FastQC was used to check the quality of the Fastq files (v0.11.2). Raw sequencing reads were aligned using the GRCh38/hg38 reference genome. GATK 3.8 best practices utilizing Sentieon’s DNASeq pipeline v201808.03 (Sentieon, San Jose, CA, USA) were used to obtain genotype calls in variant call format (VCF).

Multi-allelic sites were split using bcftools version 1.9, and then, indels were left-normalized. Quality control (QC) was carried out to exclude individuals and variants of poor sequencing quality. We removed samples with (1) a ratio of heterozygotes to non-reference homozygotes > 2.5; (2) an average read depth < 15; (3) ambiguous sex; (4) a missing genotype rate > 5%; and (5) highly related or duplicated individuals using PLINK 1.09 PI_HAT > 0.4 [15]. We removed variants that (1) did not have a GATK PASS filter value, GATK InbreedingCoef < −0.2, and GATK ExcessHet < 54; and (2) had a missing genotype rate > 5%. After QC, the number of remaining participants was 14,259.

### 2.3. Population Structure and Admixture

Population structure and admixture analysis was conducted using a set of independent common single nucleotide variants (SNVs) that had a minor allele frequency (MAF) > 0.05 and did not deviate from the Hardy–Weinberg Equilibrium (*p* > 10^−8^). Independent SNVs were extracted using linkage disequilibrium pruning (PLINK ‘--indep 50 5 1.05’), which led to 215,666 SNVs across autosomal chromosomes. The population structure was evaluated using PC-AiR [16], which accounts for relatedness, as inferred by genome-wide complex trait analysis (GCTA) [17]. For PC-AiR analysis, the 1000 Genomes Project five ancestries [18] were merged with our data. Admixture analysis was performed using ADMIXTURE [19], and ancestry groups within our cohort were determined based on the admixture fractions (details can be found in the Supplemental Materials).

### 2.4. Arrhythmia-Related Gene Panel Selection

We generated a list of candidate genes that have been associated with cardiac arrhythmias including AF. The gene panel was developed using ClinVar, Online Mendelian Inheritance in Man (OMIM), and manual curation. The ClinVar and OMIM databases were searched on 19 May 2022, using the keywords “arrhythmia” or “atrial fibrillation”. The gene panel using ClinVar and OMIM yielded 400 genes; 378 genes were from ClinVar and 22 from OMIM (not present in ClinVar). A literature search returned a total of 616 unique publications. The PubMed interface was used to remove any redundant publications, and the remaining publications were reviewed to ensure that they were related to arrhythmia. A total of 31 papers were included, adding 10 manually curated genes. In total, 410 genes were identified (Online Supplemental Table S1) encompassing 2,195,483 variants with a MAF < 0.05. The MAF distribution of these variants can be found in Supplemental Figure S1. Functional variant annotation was performed using the Ensembl Variant Effect Predictor (VEP) [20]. ClinVar (22 January 2022 version) was used to annotate variants as pathogenic (P), likely pathogenic (LP), benign (B), likely benign (LB), or variant of uncertain significance (VUS). Variants linked to arrhythmia phenotypes in ClinVar were extracted using the clinvar_CLNDN variable with a set of diagnoses shown in the Supplemental Materials. The QIAGEN clinical insight interpret (QCII) software (version 9.0.0.20220826, 10 November 2022) was independently used to compute pathogenicity and actionability based on the American College of Medical Genetics (ACMG)/Association of Molecular Pathology (AMP) 2015 guidelines [21]. Only variants that might be linked to arrhythmia and AF phenotypes were selected by QCII, which uses various databases and knowledge-based sources to infer the pathogenicity of variants. More details on the QCII parameters used can be found in the Supplemental Materials. QCII outputs the pathogenicity of each variant as P, LP, B, LB, or VUS.

### 2.5. Generation of Atrial Fibrillation Phenotypes from Electronic Medical Records (EMRs)

EMRs for 8308 participants were available based on International Classification of Diseases, 10th Revision Codes (ICD10). AF phenotype was determined using the ICD code ‘I48’. In total, only 67 AF patients were found.

### 2.6. Association Analysis

Association analysis was performed between the 1196 prioritized rare variants and AF disease status. Logistic regression was applied adjusting for sex, age, and the first 5 principal components (PCs). Single-marker (performed on the rare variants) and burden (performed on the 410 selected genes) association tests were conducted. The gene boundaries were extracted from the Ensembl genome browser (https://grch37.ensembl.org/Homo_sapiens/Info/Index, 15 September 2023). Association analysis was performed in the data as a whole (pan-ancestry). We omitted ancestry-specific analysis because of the small sample sizes of several ancestry groups. All association analyses were conducted using R software (version 4.1.1; Vienna, Austria).

### 2.7. PRS Performance

PRSs were downloaded from the PGS catalog (https://www.pgscatalog.org/; 10 October 2023). All AF PRSs with at least 20 SNVs (*N* = 10) were selected and were tested in our cohort. The list of the 10 PRSs are shown in Table 1. Logistic regression models were performed between AF and PRSs adjusting for sex, age, and 5 PCs. The odds ratio (OR) per 1 SD increase and AUC were used as performance metrics to compare PRSs.

## 3. Results

### 3.1. Population Structure and Admixture

**Figure 1:**
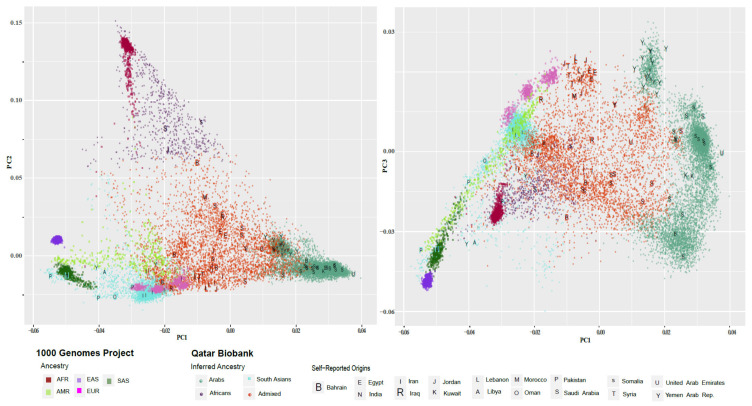
Population structure analysis in the QBB data. Population structure analysis using PC-AiR, which is a principal component analysis that accounts for relatedness among participants. The figure shows the four major ancestry groups in the QBB data inferred using ADMIXTURE. The five major ancestries in the 1000 Genomes Project are shown with QBB subpopulations: Arabs (e.g., Gulf region, Middle East, North Africa), South Asians (e.g., Iran, India), Africans, and Admixed. (**Left**) Principal component 1 (PC1) vs. principal component 2 (PC2); and (**Right**) principal component 1 (PC1) vs. principal component 3 (PC3).

PC-AiR and ADMIXTURE revealed extensive population stratification and admixture for the Middle Eastern population (Figure 1). ADMIXTURE indicated an optimal number of nine clusters based on the lowest cross-validation error. The nine clusters were merged into four clusters based on individual ancestry data in the cohort (see details in the Supplemental Materials). The four clusters of the Middle Eastern population were composed of Arabs (*N* = 6816, 47.8%), South Asians (*N* = 2620, 18.4%), Africans (*N* = 655, 4.6%), and Admixed (*N* = 4168, 29.2%). The Arab cluster encompassed individuals with origins from the Gulf (the largest group of the cluster) region as well as from other countries in the Middle East and North Africa region. The South Asian cluster included individuals with origins from Iran (the largest group of the cluster), India, and Pakistan. PC-AiR analysis, shown in Figure 1, was conducted on the QBB and the 1000 Genomes Project data combined, and survey information about the QBB’s individual origins are shown in the figure.

### 3.2. Variant Pathogenicity Classification

**Figure 2:**
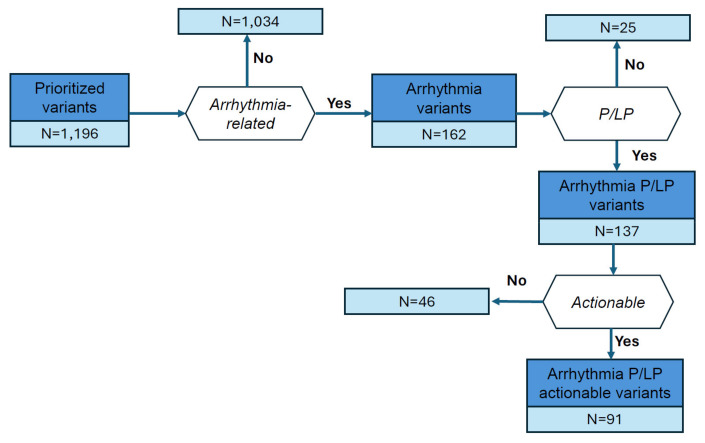
Summary of prioritized variants with respect to pathogenicity by ClinVar and QCII, and their link to arrhythmia phenotypes as defined in the Supplemental Materials.

In the 410 selected genes, we found 1196 rare variants with MAF < 0.05, classified as ‘high’ by VEP or P/LP by either ClinVar or QCII (ACMG criteria). Among these, 162 (13.55%) were previously associated with arrhythmia (AR) according to ClinVar (Figure 2). ClinVar or QCII classified 137 variants as P/LP, where 91 of these variants were within 26 unique actionable genes as per ACMG guideline (Figure 2, Online Supplemental Table S2). A total of 1030 individuals (~7%) carried at least one actionable rare variant. The percentage of AF patients among the actionable variant carriers was 4.4%.

### 3.3. Rare Clinically Relevant Arrhythmia-Related Variants in Middle Eastern Ancestral Subpopulations Compared to gnomAD Dataset

**Figure 3:**
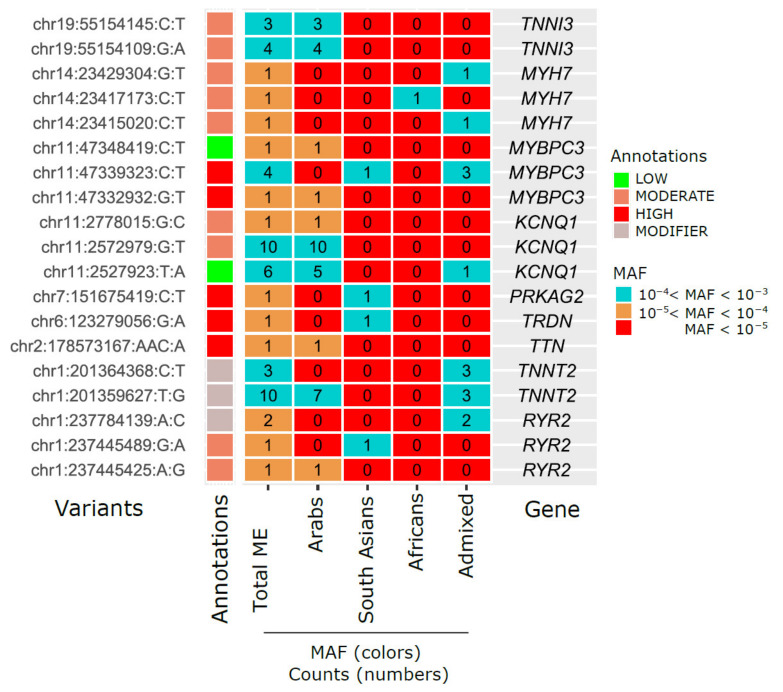
Distribution of actionable P/LP variants that are absent from gnomAD across different ancestries by MAFs.

**Figure 4:**
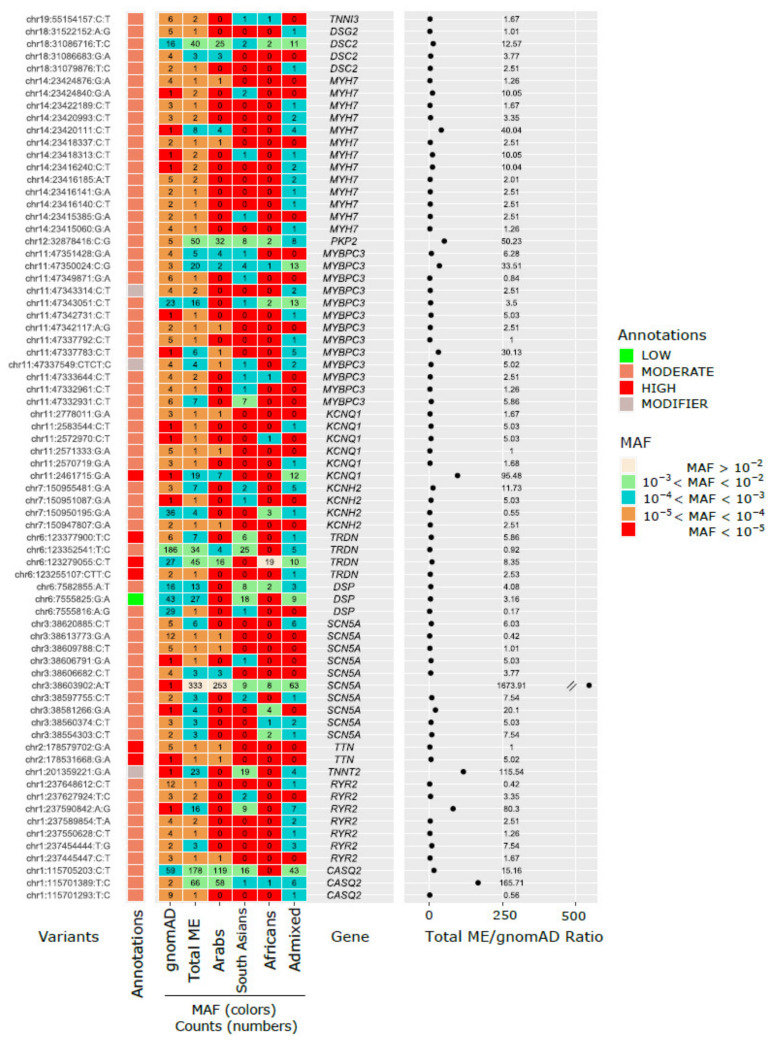
Distribution of actionable P/LP variants with frequencies more than 5-fold higher than gnomAD across different ancestries by MAFs. This figure illustrates the distribution of pathogenic/likely pathogenic (P/LP) genetic variants across various ethnic groups, with a specific focus on variants that have a frequency more than five times higher than observed in the gnomAD. The data are categorized by MAF ranges, depicted in different colors for easy reference: MAF > 10^−2^, 10^−3^ < MAF < 10^−2^, 10^−4^ < MAF < 10^−3^, 10^−5^ < MAF < 10^−4^, and MAF < 10^−5^. The ethnic groups analyzed include Middle Eastern (ME), Arab, South Asian, African, and Admixed populations. The figure also presents the Total ME/gnomAD ratio, representing the ratio of the MAFs in the ME population to the MAFs in the gnomAD dataset. The annotations indicate the impact of these variants, categorized as low, moderate, high, or modifier.

In our Middle Eastern cohort, 21% (*N* = 19) of the 91 identified rare actionable variants were absent from the gnomAD (Figure 3), and 56% (*N* = 51) exhibited more than two-fold higher MAFs (Figure 4). The MAFs of the 19 rare variants found only in the Middle Eastern population ranged from 0.000035 (1 carrier out of 14,259) to 0.00035 (10 carriers out of 14,259) (Figure 3). The variants with the highest MAFs were detected within the *KCNQ1* and *TNNT2* actionable genes. For instance, the *KCNQ1* variant rs120074186 (11:2572979:G:T, MAF = 0.000351, 10 individual carriers) was exclusively present in the Arab subpopulation, while the *TNNT2* variant (chr1:g.201359627T>G, 1:201359627:T:G, MAF = 0.000351, 10 individual carriers) was predominantly observed in the Arab and Admixed subpopulations (Figure 3).

This figure illustrates the distribution of pathogenic/likely pathogenic (P/LP) genetic variants, which are not present in the Genome Aggregation Database (gnomAD), across different ethnic groups. These variants are categorized by their MAFs, represented in distinct color codes for ranges: MAF > 10^−2^, 10^−3^ < MAF < 10^−2^, 10^−4^ < MAF < 10^−3^, 10^−5^ < MAF < 10^−4^, and MAF < 10^−5^. The ethnic groups highlighted include Middle Eastern (ME), Arab, South Asian, African, and Admixed populations. The chart provides a comparative analysis of the presence and frequency of these actionable variants in various populations.

Among the 51 actionable variants with over a two-fold higher frequency in the Middle Eastern populations compared to gnomAD, the MAFs ranged from 0.000035 (1 carrier out of 14,259) to 0.01168 (333 carriers out of 14,259). Of these, 32 variants demonstrated more than a 5-fold increase, with the highest reaching a 1674-fold elevation (Figure 4). The most prevalent variant, found within *SCN5A* (rs199473124, 3:38603902:A:T, MAF = 0.01168, 333 carriers out of 14,259), showed varying MAFs among different Middle Eastern subpopulations, with Arabs exhibiting the highest (MAF = 0.019) and South Asians the lowest (MAF = 0.00172) frequencies (Figure 4). This variant was observed once in gnomAD (Figure 4). Additionally, another variant within *SCN5A* (3:38581266:G:A) was identified only in the African subpopulation, with a MAF of 0.003, and was observed only once in gnomAD (Figure 4; Online Supplemental Table S2).

Moreover, the *TNNT2* variant rs367785431 (1:201359221:G:A, MAF = 0.0008065, 23 carriers out of 14,259) was mainly present in the South Asian (MAF = 0.004) and Admixed (MAF = 0.0005) subpopulations, showing a 115-fold increase in frequency compared to gnomAD. The *KCNQ1* variant rs1490391959 (11:2461715:G:A, MAF = 0.00067, 19 carriers) was primarily observed in the Arab (MAF = 0.0005) and Admixed (MAF = 0.001) subpopulations (Figure 4; Online Supplementary Table S2). Detailed information on actionable genes and rare variant associations with disease phenotypes is available in Online Supplemental Table S2.

### 3.4. Association of Rare Genetic Variants and AF in Middle Eastern Populations

**Figure 5:**
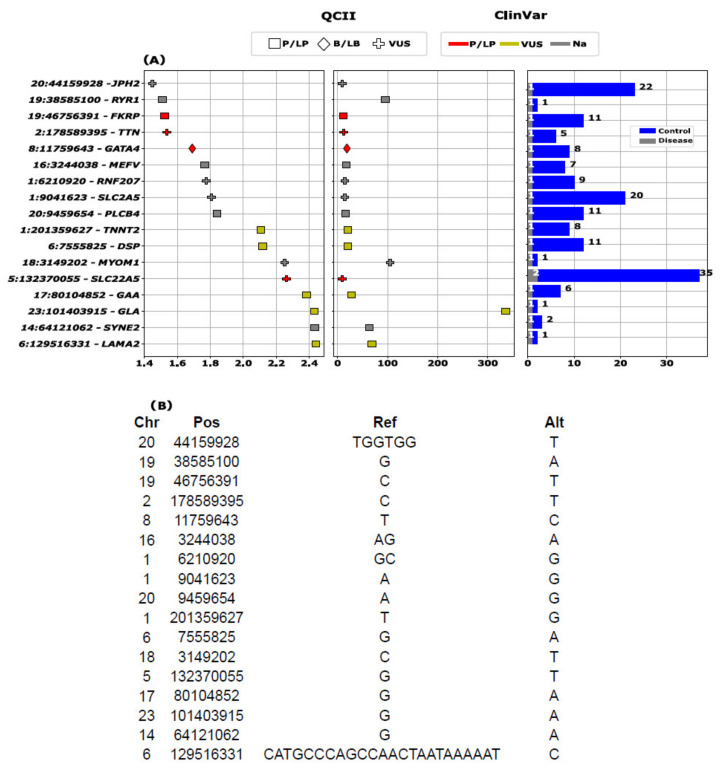
The most significant associations with atrial fibrillation. This figure illustrates a comprehensive analysis of genetic associations with atrial fibrillation (AF). (**A**) The left panel highlights the statistical significance of these associations, using a threshold of *p* < 0.05. The −log10(*p*) values are plotted on the *X*-axis, while the *Y*-axis details the variant’s chromosome position and associated gene name. The center panel displays the odds ratios (ORs) for each variant, offering insights into the strength of these associations. The right panel compares allele counts in cases versus controls, providing a clear view of variant prevalence in affected individuals versus the general population. (**B**) The table included in the figure lists the reference and alternative alleles for each variant.

Seventeen genetic variants initially exhibited potential associations (*p* < 0.05) with AF (Figure 5). A significant association was observed with a large stop loss deletion within the *LAMA2* gene locus (OR = 68.39, *p* = 3.63 × 10^−3^), specifically annotated as chr6:g.129516331_129516355delCATGCCCAGCCAACTAATAAAAAT (Figure 5). Other identified variants included those within the *SYNE2*, *GLA*, *GAA*, *SLC22A5*, *MYOM1*, *DSP*, and *TNNT2* loci (Figure 5; Online Supplemental Table S2). The *SLC22A5* variant (chr5:132370055:G:T) was absent in gnomAD but was identified in 67 individuals in our dataset, with a subset displaying AF (*N* = 2) (Figure 5). Moreover, burden tests highlighted significant associations between AF susceptibility and variants within *SCN2A* and *CPS1* (*p* = 4.9 × 10^−5^ and *p* = 9.85 × 10^−5^, respectively; Online Supplemental Material Table S3).

### 3.5. PRS Validation in Middle Eastern Population for AF

The PRS results are shown in Table 1. Five out of ten PRSs were significantly associated with AF (PGS001339, *p* = 0.025, OR = 1.32, 95% confidence interval (CI) [1.04, 1.68]; PGS000727, *p* = 0.03, OR =1.43, 95% CI [1.03, 1.97]; PGS001340, *p* = 0.032, OR = 1.31, 95% CI [1.02, 1.67]; PGS000331, *p* = 0.034, OR = 1.33, 95% CI [1.02, 1.73]; PGS000035, *p* = 0.037, OR =1.29, 95% CI [1.02, 1.65]) (Table 1) with the AUC ranging from 0.55 to 0.58 (Table 1). The OR of AF in the top PRS decile versus the remaining deciles ranged from 1.4 for PGS000035 to 1.97 for PGS001339 (Table 1). Six of the ten PRS showed OR > 1.25 (Table 1). Using the multiple testing correction (Bonferroni threshold: 0.05/103 = 0.005), no PRS remained significant.

## 4. Discussion

In this study, we analyzed WGS data from over 14,000 individuals in a Middle Eastern cohort to assess genetic susceptibility to arrhythmia phenotypes including AF. The inclusion of genes associated with a broader range of arrhythmias was considered in our study mainly for two reasons. Firstly, it was due to the potential shared genetic factors that could impact the development of AF as well as other arrhythmias. Secondly, our decision to focus primarily on AF, a specific arrhythmia phenotype, was driven by the need to enhance the overall robustness and statistical power of our analysis. By concentrating on AF, which exhibited the highest prevalence within our dataset, we were able to minimize variability and increase the likelihood of identifying genetic associations to AF.

We compared pathogenic variant frequencies across subpopulations identified within the Middle Eastern population and publicly available data (gnomAD). Our results revealed population structure was divided into four major subpopulations: Arabs (Gulf, Middle East, and North Africa), South Asians (Iranians and Indians), Africans, and Admixed. This comprehensive analysis using PC-AiR and ADMIXTURE highlights the complex population structure and admixture dynamics present within the Middle Eastern population, shedding light on the diverse genetic backgrounds and ancestral contributions shaping this region’s demographic landscape.

In total, 7% of our cohort were carriers of at least one actionable P/LP rare variant (*N* = 91 variants) identified as being associated with arrhythmias, and 4.4% of the actionable variant carriers had AF, emphasizing the potential clinical significance and prevalence of such variants within this specific population cohort. Studies including an analysis of the UK Biobank have reported 4.2% of the population with AF were P/LP carriers [22]. Rare actionable variants were examined within the Middle Eastern population, comparing their prevalence against the gnomAD dataset. Among the 91 rare arrhythmia-associated actionable variants, 56% exhibited either a greater than 2-fold MAF increase compared to gnomAD or complete absence from the dataset (21%), underscoring the potential population-specific significance. In our cohort, the variants with the highest MAFs were located in *KCNQ1* (rs120074186, MAF = 0.000351) and *TNNT2* (chr1:g.201359627T>G, MAF = 0.000351), reflecting the findings from other studies that emphasize the genetic diversity and clinical significance of AF-related variants [8]. Looking into variants with over a 2-fold higher frequency in the Middle Eastern population compared to gnomAD, which ranged from 0.000035 to 0.01168 in MAFs, revealed a diverse spectrum. Our results showed 32 variants with more than a 5-fold increase, with the most extreme increase reaching a 1674-fold peak. The variant rs199473124 within *SCN5A* (MAF = 0.01168) emerged as the most prevalent. This variant was previously associated with abnormal repolarization and AF, as reported by Boddum et al. [23]. This showcases variable frequencies among Middle Eastern subpopulations, with Arabs exhibiting the highest prevalence (MAF = 0.019) and South Asians the lowest (MAF = 0.00172), implying regional genetic heterogeneity and the complex clinical manifestations of genetic variations in the *SCN5A* gene [23].

Of particular interest were variants unique to specific subpopulations, such as the *KCNQ1* variant, rs120074186, which was exclusively present in the Arab subpopulation and linked to Long QT Syndrome [24]. The *SCN5A* variant (3:38581266:G:A) was solely identified within the African subpopulation. Furthermore, variants like rs367785431 within *TNNT2* (MAF = 0.0008065) predominantly appeared in the South Asian and Admixed subpopulations, demonstrating a 115-fold increase in frequency compared to gnomAD. Similarly, the *KCNQ1* variant, rs1490391959 (MAF = 0.00067), exhibited primary prevalence in the Arab and Admixed subpopulations, elucidating the regional clustering of these variants. Population-specific P/LP variants have been reported before by Kalayinia et al. (2023), who identified a novel stop-gain pathogenic variant in the *KCNQ1* gene in an Iranian population [25].

Exploring the potential clinical significance and prevalence of certain variants in the Middle Eastern population, we investigated their association with AF, the most common arrhythmia in this region. Eight rare actionable variants were identified to be associated with AF. This observation is particularly relevant considering a study by Al-Shamkhani et al., which revealed that AF is not only prevalent but also underdiagnosed and undertreated in the Middle East [26]. This highlights the need for targeted genetic studies and appropriate management strategies for AF in Middle Eastern populations.

A large stop-loss deletion within the *LAMA2* gene locus was significant (*p* < 0.01). The association between the *LAMA2* gene and arrhythmias has been explored in recent studies, showing that cardiac abnormalities and arrhythmias are present in a significant portion of cases with *LAMA2*-related muscular dystrophy. This highlights the potential cardiac implications of *LAMA2* gene mutations. While the specific deletion in our study was not directly linked to arrhythmias in the existing literature, the established connection between *LAMA2* mutations and cardiac abnormalities, including arrhythmias, is a crucial area of research. This relationship may provide valuable insights into how *LAMA2* mutations contribute to arrhythmia conditions, emphasizing the need for cardiac monitoring in patients with these mutations [27].

Several other variants within distinct genetic loci, including *SYNE2*, *GLA*, *GAA*, *SLC22A5*, *MYOM1*, *DSP*, and *TNNT2*, were also identified (*p* < 0.01), enriching our understanding of the genetic landscape of AF in the Middle Eastern population. Further investigation identified the *SLC22A5* variant (chr5:132370055:G:T), absent in gnomAD but present in 67 individuals within the Middle Eastern population. A subset of individuals carrying this variant had AF (*N* = 2), implying its potential relevance to AF susceptibility in this population. Studies have demonstrated the role of *SLC22A5* in metabolic carnitine deficiency and its association with cardiomyopathy features [28,29]. Additionally, Mutlu-Albayrak et al. (2015) identified the *SLC22A5* gene mutation in a family with a carnitine uptake defect, further underscoring its relevance in cardiac conditions [30]. These findings suggest that the *SLC22A5* variant might play a crucial role in the cardiac health of individuals, potentially influencing their susceptibility to conditions like AF. While direct evidence linking this variant to AF is not yet established, the noted association of *SLC22A5* with cardiac abnormalities in other studies provides a foundational basis for further investigation into its role in AF, particularly in under-represented populations such as those in the Middle East.

For common variants, we assessed the performance of PRS and quantified their predictive power. Five PRSs were significantly associated with AF, with ORs ranging from 1.29 to 1.43 and AUCs ranging from 0.55 to 0.58. However, EMRs were not available for all participants and there were missing diagnoses, which means that the controls might have contained undiagnosed AF patients. This is likely to dilute the predictive performance of the PRSs. Although these PRSs were mainly developed in individuals with European ancestries, our results suggested that they can be applied to the Middle Eastern population. It will be important to validate them in an independent Middle Eastern cohort with well-defined cases and controls and to develop ancestry-specific PRSs. Recently, several PRSs for coronary heart disease were validated in a Middle Eastern cohort [31]. To improve equitable genetic risk prediction for diverse populations, collecting more well-defined and large disease cohorts are needed.

**Table 1:** List of AF polygenic risk scores evaluated in the QBB cohort and their performance.

PRS Name *	Reference	NV	OR	Lower OR	Upper OR	*p*	AUC	OR Top Decile
PGS000016	[11]	6,730,541	1.27	0.97	1.65	8.10 × 10^−2^	0.57	1.22
PGS000035	[32]	1168	1.29	1.02	1.65	3.72 × 10^−2^	0.58	1.40
PGS000331	[12]	6,183,494	1.33	1.02	1.73	3.36 × 10^−2^	0.56	1.59
PGS000338	[33]	97	1.19	0.93	1.52	1.64 × 10^−1^	0.55	1.59
PGS000727	[34]	2,210,336	1.43	1.03	1.97	3.05 × 10^−2^	0.55	1.59
PGS001339	[35]	2142	1.32	1.04	1.68	2.50 × 10^−2^	0.56	1.98
PGS001340	[35]	2955	1.31	1.02	1.67	3.18 × 10^−2^	0.56	1.78
PGS001356	[36]	2,996,793	1.04	0.81	1.34	7.79 × 10^−1^	0.51	1.05
PGS001841	[13]	3980	1.16	0.91	1.48	2.38× 10^−1^	0.56	1.22
PGS002050	[13]	554,908	1.18	0.93	1.51	1.77 × 10^−1^	0.56	1.05

*: download from https://www.pgscatalog.org/ (accessed on 30 January 2024); NV: number of variants; AF: atrial fibrillation.

Our study encountered limitations, including the number of AF cases, which was relatively small (*N* = 67), as well as the absence of a dedicated disease-specific cohort that may not fully capture the complexities of AF. However, it is important to note that this is a reflection of the prevalence of AF in the population represented in the biobank. Despite the limited number of AF cases, the robustness of our analysis is maintained through the comprehensive genetic data available from the larger cohort. The high-quality genetic and clinical data from the 8308 patients provide a valuable context for identifying potential genetic associations with AF, even with a smaller AF cohort. Moreover, the potential for expanding our study to include the rest of the population in the biobank, with a focus on AF diagnosis, remains a viable option for future research. This broader analysis could provide additional insights into the genetic predispositions for AF in a larger, more diverse population, complementing our current findings and contributing to a more comprehensive understanding of the genetic factors influencing AF.

Despite these limitations, our research revealed significant correlations with previously unassociated functional variants, presenting new avenues for exploration in AF research. To address these constraints, we advocate for future investigations using an AF-specific cohort and the use of human-induced pluripotent stem cell-derived cardiomyocytes, as this approach could enhance our understanding of molecular mechanisms, circumventing cohort limitations and providing insights into AF pathophysiology, potentially leading to innovative therapeutic strategies.

## 5. Conclusions

This study highlights the importance of considering genetic diversity and population-specific variants in genetic risk assessment and the management of arrhythmias. This is particularly crucial for regions like the Middle East, where such studies have been limited. The identification of rare actionable variants associated with arrhythmias, particularly AF, which is prevalent yet often underdiagnosed in the Middle East, is important. The discovery of a substantial number of individuals carrying these variants indicates a higher genetic predisposition to AF in this population. Our research emphasizes the need for targeted genetic screening and personalized management strategies to improve the diagnosis, treatment, and prevention of AF and other arrhythmias in Middle Eastern populations.

## Data Availability

The data are not publicly accessible but can be obtained by following an established ISO-certified process. To access the data, you must submit a request online through the Qatar Biobank, which is subject to approval by the Qatar Biobank’s Institutional Review Board. For submission instructions, please visit https://www.qatarbiobank.org.qa/research/how-to-apply-new/ (accessed on 30 January 2024).

## Supplementary Materials

The following supporting information can be downloaded at: https://www.mdpi.com/article/10.3390/jcm13041102/s1, Figure S1: Minor allele frequency distribution in the selected variants. The *x*-axis represents the minor allele frequency (MAF) and the the *y*-axis represents the percentage of each MAF bin.; Online Supplemental Table S1: Gene panel selection details; Online Supplemental Table S2: The list of all 1196 variants identified as P/LP by either ClinVar or QCII, or having a ‘High’ impact by VEP; Online Supplemental Table S3: Association results for the burden test with AF.

## List of Abbreviations

ACMG	American college of medical genetics
AF	Atrial fibrillation
AR	Arrhythmia
AUC	Area under the receiver operating curve
B	Benign
EMR	Electronic medical record
GWAS	Genome-wide association study
ICD10	International classification of diseases, 10th revision codes
LB	Likely benign
LP	Likely pathogenic
MAF	Minor allele frequency
OMIM	Online mendelian inheritance in man
OR	Odds ratio
P	Pathogenic
PC	Principal component
PRS	Polygenic risk score
QBB	Qatar biobank
QC	Quality control
QCII	QIAGEN clinical insight interpret
QGP	Qatar genome program
SD	Standard deviation
SNV	Single-nucleotide variant
VCF	Variant call format
VEP	Ensembl Variant Effect Predictor
VUS	Variant of uncertain significance
WGS	Whole-genome sequencing
