# Supplementary material for: Genetic Susceptibility to Arrhythmia Phenotypes in a Middle Eastern Cohort of 14,259 Whole-Genome Sequenced Individuals
Source: J Clin Med. 2024 Feb 15;13(4):1102. doi: 10.3390/jcm13041102 (PMC10888535; doi:10.3390/jcm13041102)
Supplement: Supplementary file 1 [file jcm-13-01102-s001.zip › jcm-2850970-SI.pdf]

Supplemental Material

Genetic Susceptibility to Arrhythmia Phenotypes in a Middle Eastern Cohort of 14,259 Whole-Genome Sequenced Individuals

Fatima Qafoud [1], Mohamed Elshrif [2], Khalid Kunji [2], Asma Althani [1], Amar Salam [3], Jassim Al Suwaidi [4], Nidal Asaad [4], Dawood Darbar [5] and Mohamad Saad [2,*]

[1] College of Health Sciences, Qatar University, Doha P.O. Box 2713, Qatar; fkafood@hotmail.com (F.Q.); aaja@qu.edu.qa (A.A.)
[2] Qatar Computing Research Institute, Hamad Bin Khalifa University, Doha P.O. Box 5825, Qatar; melshrif@hbku.edu.qa (M.E.); kkunji@hbku.edu.qa (K.K.)
[3] Department of Cardiology, Al-Khor Hospital, Hamad Medical Corporation, Doha P.O. Box 3050, Qatar; asalam@hamad.qa
[4] Heart Hospital, Hamad Medical Corporation, Doha P.O. Box 3050, Qatar; jalsuwaidi@hamad.qa (J.A.S.); nasaad@hamad.qa (N.A.)
[5] Division of Cardiology, Department of Medicine, University of Illinois Chicago, Chicago, IL 60612, USA; darbar@uic.edu
* Correspondence: msaad@hbku.edu.qa; Tel.: +974-4454-7746

Population structure, admixture, and relatedness

Admixture analysis was performed using ADMIXTURE. Nine clusters were assigned using the lowest cross-validation error. An individual will be directly assigned to a cluster if ancestry fraction is greater than 0.5. The remaining individuals will be assigned to a group called Admixed. Leveraging survey information about individual origins in the cohort, five clusters were Arabs, three clusters were South Asians, and one cluster was African. Arab clusters were merged into one cluster by summing all five ancestry fractions. If the sum of the ancestry fractions was greater than 0.8, the individual is assigned to the Arabs cluster, and otherwise to the Admixed cluster. South Asians clusters were also merged into one cluster and similar approach was used.

Whole genome sequencing quality control for sex ambiguity

Ambiguous sex was determined as follows: for females, an individual was removed if plink F value was below mean(F[F<0.3]) + 3SD (F[F<0.3]), and for males, an individual was removed if plink F value was higher than mean(F[F>0.7]) - 3SD (F[F>0.7]).

Generation of phenotypes from ClinVar diagnosis

The following keywords were used to search for the diagnosis in the EMR data and Clinvar_ CLNDN, respectively: Atrial fibrillation and Atrial flutter, Arrhythmia, Palpitation, Tachycardia, Bradycardia, Heartbeat, Ventricular premature, Ventricular fibrillation, Long QT, Atrioventricular block, Trifascicular block, Bifascicular block, bundle branch block, Sick sinus syndrome, Premature depolarization, Syncope and collapse, iii) Cardiomyopathy, Arrhythmogenic.

QIAGEN clinical insight interpret (QCII) filters:

The initial rare variants were filtered as follows: i) we kept variants that are known or predicted to affect Arrhythmia: Arrhythmia, arrhythmogenic BiMVP syndrome (Arrhythmogenic bileaflet mitral valve prolapse syndrome), arrhythmogenic left ventricular cardiomyopathy (Left ventricular arrhythmogenic cardiomyopathy)arrhythmogenic right ventricular cardiomyopathy 10 (Arrhythmogenic right ventricular dysplasia familial 10) , arrhythmogenic right ventricular cardiomyopathy 11 (Arrhythmogenic right ventricular dysplasia familial 11)), arrhythmogenic right ventricular cardiomyopathy 12 (Arrhythmogenic right ventricular dysplasia familial 12) , arrhythmogenic right ventricular cardiomyopathy 14 (Familial arrhythmogenic right ventricular dysplasia type 14) , arrhythmogenic right ventricular cardiomyopathy 6 (Arrhythmogenic right ventricular cardiomyopathy type 6),

arrhythmogenic right ventricular cardiomyopathy 9 (Arrhythmogenic right ventricular dysplasia familial 9) , arrhythmogenic right ventricular cardiomyopathy type 1 (Familial arrhythmogenic right ventricular dysplasia type 1) , arrhythmogenic right ventricular cardiomyopathy type 3 (Familial arrhythmogenic right ventricular dysplasia type 3), arrhythmogenic right ventricular cardiomyopathy type 4 (Familial arrhythmogenic right ventricular dysplasia type 4), arrhythmogenic right ventricular cardiomyopathy with left ventricle involvement (Arrhythmogenic right ventricular cardiomyopathy with left ventricular involvement), arrhythmogenic right ventricular cardiomyopathy/dysplasia (Arrhythmogenic right ventricular cardiomyopathy), arrhythmogenic right ventricular cardiomyopathy/dysplasia with diffuse left ventricular involvement (Arrhythmogenic right ventricular dysplasia with diffuse left ventricular involvement), arrhythmogenic right ventricular dysplasia 5 (Familial arrhythmogenic right ventricular dysplasia type 5) , arrhythmogenic right ventricular dysplasia type 2 (Familial arrhythmogenic right ventricular dysplasia type 2), arrhythmogenic right ventricular dysplasia type 8 (Familial arrhythmogenic right ventricular dysplasia type 8), atrial bradyarrhythmia (Bradycardia of atrium), atrial conduction (Conduction of atrium) , atrial contraction (Contraction of atrium) , atrial depolarization (Depolarization of atrium) , atrial development (Development of atrium) , atrial dilation (Abnormal morphology of dilated atrium) , atrial dysplasia (Abnormal morphology of atrium) , atrial dysrhythmia (Arrhythmia of atrium) , atrial ectopic rhythm (Ectopic atrial rhythm) , atrial ectopic tachycardia (Ectopic atrial tachycardia), atrial fibrillation with bradyarrhythmia (Autosomal dominant sick sinus syndrome type 2) (affected genes: HCN4), atrial tachyarrhythmia (Tachycardia of atrium) , autosomal dominant myocardiopathy (Autosomal dominant cardiomyopathy) , bradyarrhythmia (Bradycardia) , bradyarrhythmia absoluta (Absolute bradycardia), bradyarrhythmia and sinus pause (Sinus pause and bradycardia), bradyarrhythmic disorder (Bradycardia) , bradyarrhythmic disorder of atrial heart chamber (Bradycardia of atrium), bradycardia with non-compaction cardiomyopathy and aortic dilatation (Bradycardia with ventricular noncompaction and aortic dilatation), bradycardia-induced acquired long QT syndrome (Bradycardia-associated form of long QT syndrome) (affected genes: KCNH2), bradycardia-induced long QT syndrome (Long QT syndrome 2) , bradycardia-tachycardia syndrome (Tachycardia bradycardia syndrome), congenital long-QT syndrome 3 (Long QT syndrome 3) , congenital myocardiopathy (Congenital cardiomyopathy) , function threatening type 2 congenital long-QT syndrome (Severe long QT syndrome 2), incessant infant ventricular tachyarrhythmia (Incessant infant ventricular tachycardia), late-onset myocardiopathy (Late-onset cardiomyopathy) , late-onset supraventricular tachyarrhythmia (Late-onset supraventricular tachycardia), life-threatening long-QT syndrome (Severe long-QT syndrome) , long-QT syndrome 6 (Long qt syndrome type 6), long-QT

syndrome 7 (Andersen syndrome) , long-QT syndrome 9 (Long qt syndrome type 9) , long-QT syndrome type 10 (Long-QT syndrome 10) , malignant ventricular tachyarrhythmia (Malignant ventricular tachycardia), mild grade myocardiopathy (Mild cardiomyopathy), mild grade recurrent tachyarrhythmia (Mild recurrent tachyarrhythmia), mild grade tachyarrhythmia (Mild tachycardia), mild type 2 congenital long-QT syndrome (Mild long QT syndrome 2) (affected genes: KCNH2), mitochondrial myocardiopathy with biventricular fibro-adipose replacement (Mitochondrial cardiomyopathy with biventricular fibro-adipose replacement), moderate type 2 congenital long-QT syndrome (Moderate long QT syndrome 2), modest myocardiopathy (Moderate cardiomyopathy) , myocardiopathy (Cardiomyopathy), myocardiopathy of cardiac ventricle (Cardiomyopathy of heart ventricle), myocardiopathy of left ventricular cavity (Cardiomyopathy of left ventricle) , myocardiopathy of right ventricle of heart (Right ventricular cardiomyopathy) , myocardiopathy progression (Progressive cardiomyopathy) (affected genes: DAG1, DHX36, DICER1, MED30, MYH6, TNFSF12), reversible myocardiopathy (Reversible cardiomyopathy), serious myocardiopathy (Severe cardiomyopathy), tachyarrhythmia of atrial heart chamber (Tachycardia of atrium) , tachyarrhythmia of cardiac ventricle (Ventricular tachycardia) , tachycardia paroxysmal (Paroxysmal tachycardia), tachycardia-bradycardia (Tachycardia bradycardia syndrome), type 2 congenital long-QT syndrome (Long QT syndrome 2), ventricular ablation (Cell death of heart ventricle), ventricular arrhythmias (Arrhythmia of heart ventricle), ventricular cardiac muscle tissue morphogenesis (Morphogenesis of cardiac muscle tissue in heart ventricle), ventricular cardiac remodeling (Ventricular remodeling) , ventricular cardiomyocytes ablation (Cell death of ventricular myocytes) , ventricular cardiomyocytes activation (Activation of ventricular myocytes) , ventricular cardiomyocytes afterdepolarization (Afterdepolarization of ventricular myocytes), ventricular cardiomyocytes conduction (Conduction of ventricular myocytes) , ventricular cardiomyocytes contraction (Contraction of ventricular myocytes) , ventricular cardiomyocytes differentiation (Differentiation of ventricular myocytes), ventricular cardiomyocytes disassociation (Dissociation of ventricular myocytes), ventricular cardiomyocytes division (Cell division of ventricular myocytes) , ventricular cardiomyocytes hypoxia-reperfusion (Ischemia reperfusion of ventricular myocytes), very severe congenital long-QT syndrome 3 (Severe long QT syndrome 3) , younger onset congenital long-QT syndrome 3 (Early-onset long QT syndrome 3) or diseases consistent with these phenotypes.

ii) Kept exonic variants (up to 20 bases into the intron) that are experimentally observed to be associated with a phenotype: Pathogenic, Possibly Pathogenic, Unknown Significance, Possibly Benign, Benign OR Disease-associated according to HGMD OR Disease-associated according to CLINVAR OR Clinically

relevant variants from CentoMD OR established gain of function in the literature OR gene fusions OR inferred activating mutations by Ingenuity OR predicted gain of function by BSIFT OR in a microRNA binding site OR overlap with copy number gains OR Frameshift, in-frame indel, or stop codon change OR Missense OR predicted deleterious by having CADD score > 10.0 ORsplice site within 10 bases into intron OR predicted to disrupt splicing by MaxEntScan OR in an ENCODE transcription factor binding site overlapping promoter region OR in enhancer.

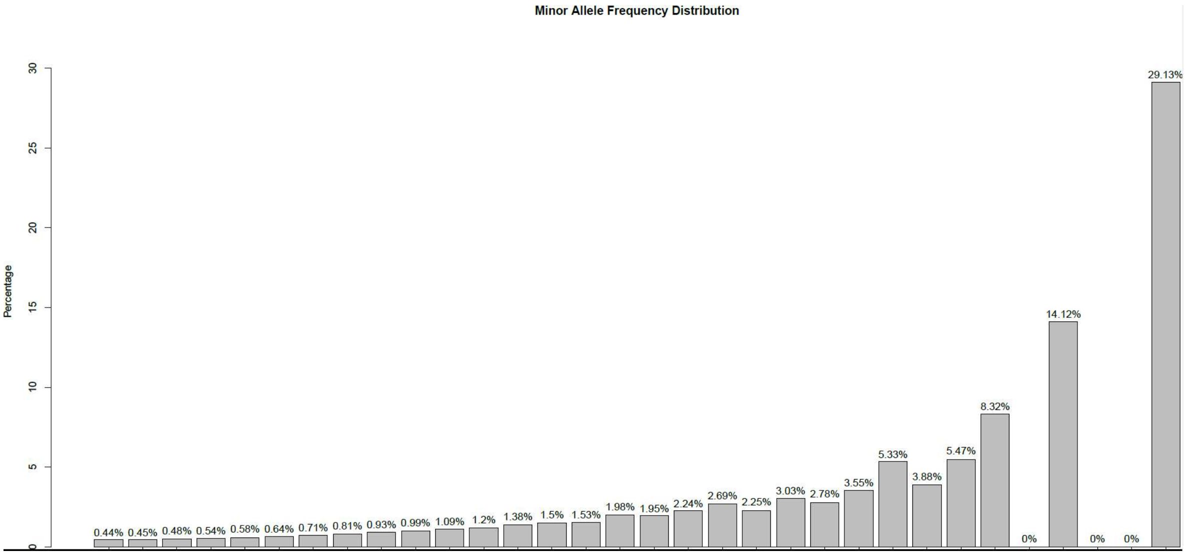

Supplemental Figure S1: Minor allele frequency distribution in the selected variants. The x-axis represents the minor allele frequency (MAF) and the the y-axis represents the percentage of each MAF bin

Online Supplemental Table S1: Gene panel selection details
4ClinVar or QCII, or having a 'High' impact by VEP within the 410 selected genes
Online Supplemental Table S3: Association results for the burden test with AF
